# Technical variability across the 450K, EPICv1, and EPICv2 DNA methylation arrays: lessons learned for clinical and longitudinal studies

**DOI:** 10.1186/s13148-024-01761-4

**Published:** 2024-11-22

**Authors:** Alexandre A. Lussier, Isabel K. Schuurmans, Anna Großbach, Julie Maclsaac, Kristy Dever, Nastassja Koen, Heather J. Zar, Dan J. Stein, Michael S. Kobor, Erin C. Dunn

**Affiliations:** 1 Psychiatric and Neurodevelopmental Genetics Unit, Center for Genomic Medicine, Massachusetts General Hospital, Boston, MA USA; 2 Department of Psychiatry, Harvard Medical School, Boston, MA USA; 3 Stanley Center for Psychiatric Research, The Broad Institute of Harvard and MIT, Cambridge, MA USA; 4 Department of Child and Adolescent Psychiatry/Psychology, Erasmus MC University Medical Center Rotterdam, Rotterdam, The Netherlands; 5 School of Mathematical and Statistical Sciences, University of Galway, Galway, Ireland; 6 The SFI Centre for Research Training in Genomics Data Science, Dublin, Ireland; 7 Department of Medical Genetics, Faculty of Medicine, University of British Columbia, Vancouver, BC Canada; 8 British Columbia Children’s Hospital Research Institute, University of British Columbia, Vancouver, BC Canada; 9 Centre for Molecular Medicine and Therapeutics, University of British Columbia, Vancouver, BC Canada; 10 SAMRC Unit on Risk and Resilience in Mental Disorders, Department of Psychiatry and Neuroscience Institute, University of Cape Town, Cape Town, South Africa; 11 Department of Pediatrics and Child Health, Red Cross War Memorial Children’s Hospital, University of Cape Town, Cape Town, South Africa; 12 South African Medical Research Council (SAMRC) Unit on Child and Adolescent Health, University of Cape Town, Cape Town, South Africa; 13 Edwin S.H. Leong Centre for Healthy Aging, University of British Columbia, Vancouver, BC Canada; 14 Department of Sociology, College of Liberal Arts, Purdue University, West Lafayette, IN USA

**Keywords:** Epigenetics, Drakenstein Child Health Study, DNA methylation, Illumina arrays, Longitudinal, Reproducibility

## Abstract

DNA methylation (DNAm) is the most commonly measured epigenetic mechanism in human populations, with most studies using Illumina arrays to assess DNAm levels. In 2023, Illumina updated their DNAm arrays to the EPIC version 2 (EPICv2), building on prior iterations, namely the EPIC version 1 (EPICv1) and 450K arrays. Whether DNAm measurements are stable across these three generations of arrays has yet not been investigated, limiting the ability of researchers—especially those with longitudinal data—to compare and replicate results across arrays. Here, we present results from a study of 30 child participants (15 male; 15 female) from the Drakenstein Child Health Study, who had DNAm measured on all three of the latest arrays: 450K, EPICv1, and EPICv2. Using these data, we created an annotation of probe quality across arrays, which includes the intraclass correlations, interquartile ranges, correlations, and array bias (i.e., the extent to which DNAm levels were explained by array type) of all CpGs. We also present results from an analysis of sex differences, where we found that CpGs with lower replicability across arrays had higher array-based variance, suggesting this variance metric help guide replication efforts. We also showed that epigenetic age estimates across arrays were more stable when using the principal component versions of epigenetic clocks. Ultimately, this collection of results provides a framework for investigating the replicability and longitudinal stability of epigenetic changes across multiple versions of Illumina DNAm arrays.

**Supplementary Information:**

The online version contains supplementary material available at 10.1186/s13148-024-01761-4.

## Introduction

In recent years, epigenetic mechanisms have emerged as a promising avenue to explain associations between genetic factors, environmental exposures, and health outcomes [1, 2]. One such epigenetic mechanism is DNA methylation (DNAm) [3], which involves the addition of methyl molecules to specific DNA base pairs (at cytosine-guanine dinucleotides; CpGs) to tag, stabilize, or regulate genomic regions [4]. DNAm levels can be quantified in various tissues, including the brain, blood, and saliva, and are known to associate with several health risk factors, including prenatal exposure to smoking [5], and adverse health outcomes (e.g., mortality rates [6]). Such associations are often identified in epidemiological cohort studies [7]. For a full understanding of the role of DNAm, researchers need tools that can reliably measure DNAm on an extensive scale across large proportions of the genome.

The gold standard for DNAm assessment is whole-genome bisulfite sequencing (WGBS), which achieves single-nucleotide precision and encompasses approximately 95% of all CpGs in the human genome—covering around 28 million loci [8]. Despite its high accuracy and comprehensive coverage, WGBS is costly, limiting its practicality for large-scale, population-based studies. To bridge this gap, Illumina Infinium ® produced a series of BeadChip microarrays. In 2008, they introduced the *HumanMethylation27 BeadChip*, which covered approximately 27,000 CpGs. The coverage extensively expanded in 2011 with the *HumanMethylation450 BeadChip* (450K), measuring over 485,000 CpGs [9], and in 2016, with the release of the *HumanMethylationEPIC BeadChip* (EPICv1 or 850 K), measuring over 850,000 CpGs [10]. Most recently in 2023, the *HumanMethylationEPIC v2.0 BeadChip* (EPICv2 or 900 K) was launched [11]. This latest version incorporates an additional 186,000 CpGs informed by cancer research, enriching its content with enhancers, CTCF-binding sites, CpG islands, and improved copy number variation detection for clinical applications.

The evolution of DNAm arrays can impact the comparability of data within and across studies. Arrays, after all, have undergone substantial change over time, including the removal of poor-quality sites and the addition of new probes. While these changes generally reflect an optimization process, they also present unique challenges, particularly for studies attempting to replicate their findings and longitudinal cohort studies that transition between array versions due to ongoing data collection and processing. A small number of empirical studies have performed back-to-back comparisons of sequentially developed arrays. These comparisons have consistently showed high correlations at the sample level between the 450K and EPICv1 arrays [12–14] and high reproducibility across different tissue types within the EPICv2 array [11, 15]. Yet, notable discrepancies in DNAm levels between arrays are observed at individual CpG sites—evidenced in both human cord [12] and peripheral blood [14]—which poses potential risks for consistency in longitudinal studies and increased difficulty for replication within and across epigenetic studies.

To our knowledge, no studies have compared the 450K, EPICv1, and EPICv2 arrays within the same population-based cohort, even though the development of these chips within about a decade means they are likely to be used in multi-decade longitudinal studies. Analyzing these arrays collectively in a single study will pave the way for harmonization strategies in longitudinal datasets, as it would allow scientists to differentiate technical differences from longitudinal changes in DNAm levels. These analyses can also inform clinical or direct-to-consumer applications of epigenetic arrays, as for-profit companies have begun selling DNAm assessment tools, especially epigenetic clock derivation measures. Thus, researchers and companies alike will benefit from a better understanding of DNAm measurement consistency and comparability across generations of arrays.

The current study aimed to address this gap, first by undertaking a comprehensive examination of DNAm comparability and stability across three generations of arrays within the same population-based cohort. Second, we assessed the consistency of associations derived from widely used epigenetic clocks to ascertain the stability of markers into epigenetic age estimations, one of the most popular uses of DNAm data in human populations. Finally, we provide recommendations for longitudinal studies, aimed at facilitating the integration of epigenetic datasets across different generations of arrays.

## Materials and methods

### Study population

Participants were children from the Drakenstein Child Health Study (DCHS). The DCHS is a longitudinal birth cohort study in the Drakenstein sub-district of the Western Cape, South Africa, a peri-urban area about 60 km outside of Cape Town [16]. From March 2012 to March 2015, 1137 pregnant women (with 1143 live births) were recruited at 20–28 weeks’ gestation from two primary care clinics in the Drakenstein sub-district in Paarl [17]. The first site (TC Newman) serves a predominantly mixed ancestry community, while the second site (Mbekweni) serves primarily a black African ancestry community. Please note that ancestry was self-reported, not quantified by genetic metrics. In the context of the DCHS, self-reported ancestry is not reported to reify social categories, but rather to contribute to the literature on ongoing socioeconomic disparities. Overall, the DCHS cohort is representative of many peri-urban regions in South Africa and other low- and middle-income countries, with lower socioeconomic status and maternal educational attainment and higher rates of psychosocial risk factors than other population-based studies [16]. The cohort, as well as inclusion and exclusion criteria, have previously been comprehensively described [16, 17].

### Analytic sample

We randomly selected 15 male and 15 female children from the subset of DCHS participants who provided a whole blood sample at approximately 5 years of age. Among this random selection, we ensured the male versus female samples were not biased by checking a priori if the random selection of participants were not significantly different (*p* < 0.05) with respect to the following variables, which are known to influence DNAm levels: (1) self-reported ancestry; (2) maternal-reported antenatal maternal education (socioeconomic position metric); or (3) maternal-reported exposure to prenatal smoking (Table S1). Whole blood was collected by venipuncture and transported to the research laboratory in Cape Town on ice on the day of sampling and stored at −80C.

**Table 1:** Summary of the analytic sample

	Total DCHS sample	DCHS analytic subsample	*p* value
*N*	1143	30	
Sex			1.00
Female	48.7%	50.0%	
Male	51.3%	50.0%	
Ancestry			0.027
Black African	55.3%	33.3%	
Mixed Ancestry	44.6%	66.7%	
Education			0.08
Less than secondary	60.8%	43.3%	
Secondary or greater	39.2%	56.7%	
Prenatal smoking			0.42
Exposed	71.7%	63.3%	
Unexposed	28.3%	36.7%	
Age at DNAm collection (years)		5.06 (0.054)	
Maternal age at birth (years)	26.9 (5.72)	26.7 (6.62)	0.88
Birthweight (grams)	3019 (611)	3121 (667)	0.41
Parity (number)	1.04 (1.06)	1 (1.14)	0.85
Gestational age (weeks)	38.4 (2.66)	38.5 (2.62)	0.87

*Chi-squared tests were used for categorical variables. *T*-tests were used for continuous variables. Mean and standard deviation are shown for continuous variables

### DNA methylation data generation, processing, and normalization

DNA methylation was measured from whole blood collected at 5 years of age [18] using the three most recent generations of the Illumina DNAm array. DNA was extracted from whole blood collected at 5 years of age using the Qiagen DNeasy DNA Blood & Tissue kit (Qiagen, USA), and 750 ng of DNA was bisulfite-converted according to the manufacturer’s instructions using the Zymo EZDNA bisulfite conversion kit (Zymo Research, USA).

Genome-wide DNAm was measured for each sample using the (1) Illumina Infinium HumanMethylation450 BeadChip (450K; 485,577 probes), (2) the Illumina MethylationEPIC BeadChip (EPICv1; 866,552 probes), and (3) HumanMethylationEPIC v2.0 BeadChip (EPICv2; 937,690 probes). As the 450K and EPIC arrays have a capacity of 12 and 8 samples per array, respectively, we filled the remaining 6 and 2 positions with technical replicate (meaning duplicate) samples. Specifically, we included six replicates to the 450K arrays (3 chips × 12 samples = 36 positions) and two replicates to the EPICv1 and EPICv2 arrays (4 chips × 8 samples = 32 positions). The samples used as technical replicates were selected randomly from all available samples and two were used across all array versions.

Raw DNAm data were processed using the *meffil 1.3.4* pipeline [19]. No samples were removed due to > 10% of probes having detection *p* values > 0.01 or bead numbers < 3. We removed probes with a detection *p* value > 0.01 or bead number < 3 in more than 20% of samples, which resulted in the removal of 237 probes from the 450K array, 1141 from EPICv1, and 1113 from EPICv2. We used a more lenient threshold for probe removal compared to established practices [19], so we could provide a broader picture for readers. Following these pre-processing steps, we normalized the data using functional normalization, a between-array normalization method that minimizes *technical* variation by regressing out the variability explained by array control probes [20].

We processed the DNAm data in two ways to better understand how this processing step might influence the comparability of data between arrays. First, we processed data from each array separately, resulting in three separate datasets, one for each array. Second, we processed data all together, resulting in a single dataset composed of 369,639 CpGs present on all three arrays. Replicates were removed for primary analyses.

### Analyses

All analyses were completed using R version 3.6.1. Probe annotations were obtained from the manifest files available in *meffil* 1.3.4. Specific analyses to supplement these annotations and investigate the reliability of probes across arrays are described below.

#### Intraclass correlations

The reliability of CpG-level data can be influenced by both person-to-person (i.e., biological differences between people) and technical variation in measurement. Such reliability is often assessed by calculating intraclass correlation coefficients (ICC), a statistic using pairs of duplicate samples to quantify biological variability compared to the total variability (biological and technical variation). Here, we calculated the ICC for each CpG using the repicc() function from the *ENMix* 1.37.04 package. Specifically, we analyzed the two replicate samples that were present on all three of the 450K, EPICv1, and EPICv2 arrays. To ensure comparability across arrays, the other four other technical replicates on the 450K array were omitted from this analysis.

#### Interquartile range (IQR)

We calculated the IQR for each CpG using the 30 samples available on each array (replicates removed) with the rowIQRs() function from the matrixStats 0.61.0 package. Here, we considered an IQR > 0.01 as representative of measurable variability for a given CpG (i.e., 1% spread in DNAm levels between the 25th and 75th percentiles). As mean differences may be difficult to identify or too small to interpret in CpGs with low variability, IQR can help determine whether a given CpG would be informative in epigenome-wide analyses. We selected this threshold as it represents a small variance in DNAm that could be detected in most epigenome-wide studies.

#### Array-level bias

We estimated array-level bias for each CpG, using an ANOVA of array effects corrected for repeated measures from the same participant (formula: *aov(DNAm* ~ *array* + *SampleID)*), to determine the extent to which DNAm levels could be explained by array. We further quantified array differences using post hoc Tukey honestly significant difference (HSD) tests, which compared mean differences in DNAm levels between arrays.

#### DNAm quantitative trait loci (mQTL)

We annotated mQTLs using The Genetics of DNA Methylation Consortium (GoDMC) database, a large-scale GWAS of blood DNAm from 32,851 European participants [21]. *Cis*-mQTLs were defined as having a SNP with a p < 1 × 10^–8^ located < 1 Mb of the locus; *trans*-mQTLs had a SNP with a *p* < 1 × 10^–14^ located > 1 Mb from the locus.

#### Stability of associations with sex

We investigated the stability of associations between sex and DNAm across arrays using the normalized data processed together without replicates (369,639 CpGs for 30 samples on 3 arrays). We selected genetic sex for these epigenome-wide analyses because associations with DNAm are likely more robust and replicable than environmental or psychosocial measures [22]. Analysis of these data would be comparable to analyses combining data across multiple generations of arrays to maximize statistical power. We approached these analyses in two ways, both using the lmFit() function from the *limma* 3.40.6 package.

We investigated each array independently (369,639 CpGs; 30 samples/array) and assessed the overlapping associations across analyses by (1) identifying which associations were shared across array based on FDR < 0.05 and *p* < 1 × 10^–8^ thresholds, (2) comparing mean DNAm differences between males and females (∆beta) for CpGs passing *p* < 1 × 10^–8^, and (3) estimating the Pearson correlation between effect estimates. By performing these analyses, we aimed to determine the extent to which array differences could mask associations between sex and DNA methylation. All analyses were corrected for the following covariates, which have previously been associated with DNAm levels: collection site (due to robust socio-demographic and socioeconomic differences between collection sites), birthweight, gestational age in weeks, maternal-reported prenatal exposure to smoking, maternal age at birth, parity, and cell type proportions estimated using the Houseman method [23].

#### Epigenetic clocks

We calculated epigenetic age for all samples using seven current epigenetic clocks: (1) original Horvath clock (Horvath1) [24]; (2) Horvath skin & blood clock (Horvath2) [25]; (3) Hannum clock [26]; (4) PhenoAge [27]; (5) GrimAge1 [28]; (6) GrimAge2 [29]; and (7) DunedinPACE (30). Epigenetic age estimates were calculated for each array separately, using all available probes for a given array. We also calculated the principal component versions of all clocks except DunedinPACE and GrimAge2, due to data and code unavailability (31). Principal component clocks leverage principal component analysis (PCA) to refine epigenetic clocks. By consolidating data from many CpG sites into key features, rather than using DNAm levels themselves, PCA captures the core aging signal while minimizing noise from individual CpG measurements. Thus, the PCA approach to deriving epigenetic clocks can enhance the reliability and accuracy of biological age estimations.

We assessed within-person differences in epigenetic age estimates across arrays using Pearson correlations. We also extracted CpGs along with their corresponding weights in the epigenetic age algorithm for each clock to assess the relative impact of missing CpGs for each array. We first normalized the weights of all CpGs involved in the clock so together they summed to 100%. Subsequently, we assessed the proportion of this sum score represented by the missing CpGs. Of note, we could not estimate epigenetic age using the DunedinPoAm clock [32] for EPICv2 samples due to data unavailability and package options.

## Results

### Overview of the analytic sample

We first determined whether our sample was representative of the full DCHS cohort (Table 1). There were no significant differences between our analytic sample and DCHS participants with regards to sex, prenatal exposure to smoking, maternal education, maternal age at child birth, gestational age of baby at delivery, or parity. However, participants in our analytic subsample were more likely to have mixed ancestry (*p* = 0.027) and had nominally higher education (*p* = 0.08), meaning this subset may not be fully comparable to the entire DCHS sample. We also compared males and females within our subsample to determine whether demographic differences might influence sex-specific associations with DNAm. There were no significant differences between socio-demographic characteristics for male or female participants, maternal-reported ancestry, prenatal exposure to smoking, maternal education or age at birth, gestational age at delivery, parity, or age at DNAm measurement (Table S1). Males had nominally higher birthweights than females (*p* = 0.051).

### SNP probe reliability

We first matched samples across arrays based on the 57 SNP probes present across all three arrays. These probes are typically used to uniquely identify samples based on genetic variation, allowing for samples to be matched across arrays. However, the EPICv2 samples clustered separately from their 450K and EPICv1 counterparts when using this set of 57 SNPs, resulting in multiple sample mismatches (Figure S1A). Upon further investigation, we identified 21 SNP probes on the EPICv2 that showed different probe sequences, color channels, and/or a next base from previous generations of the array (Table S2). Removing these 21 probes caused the EPICv2 samples to accurately cluster (Figure S1B). Based on these results, we recommend removing the 21 mismatched SNP probes listed as having any bad metric (“bad_metric” column) in Table S2 when matching samples across arrays.

### Annotation of CpGs

**Fig. 1:**
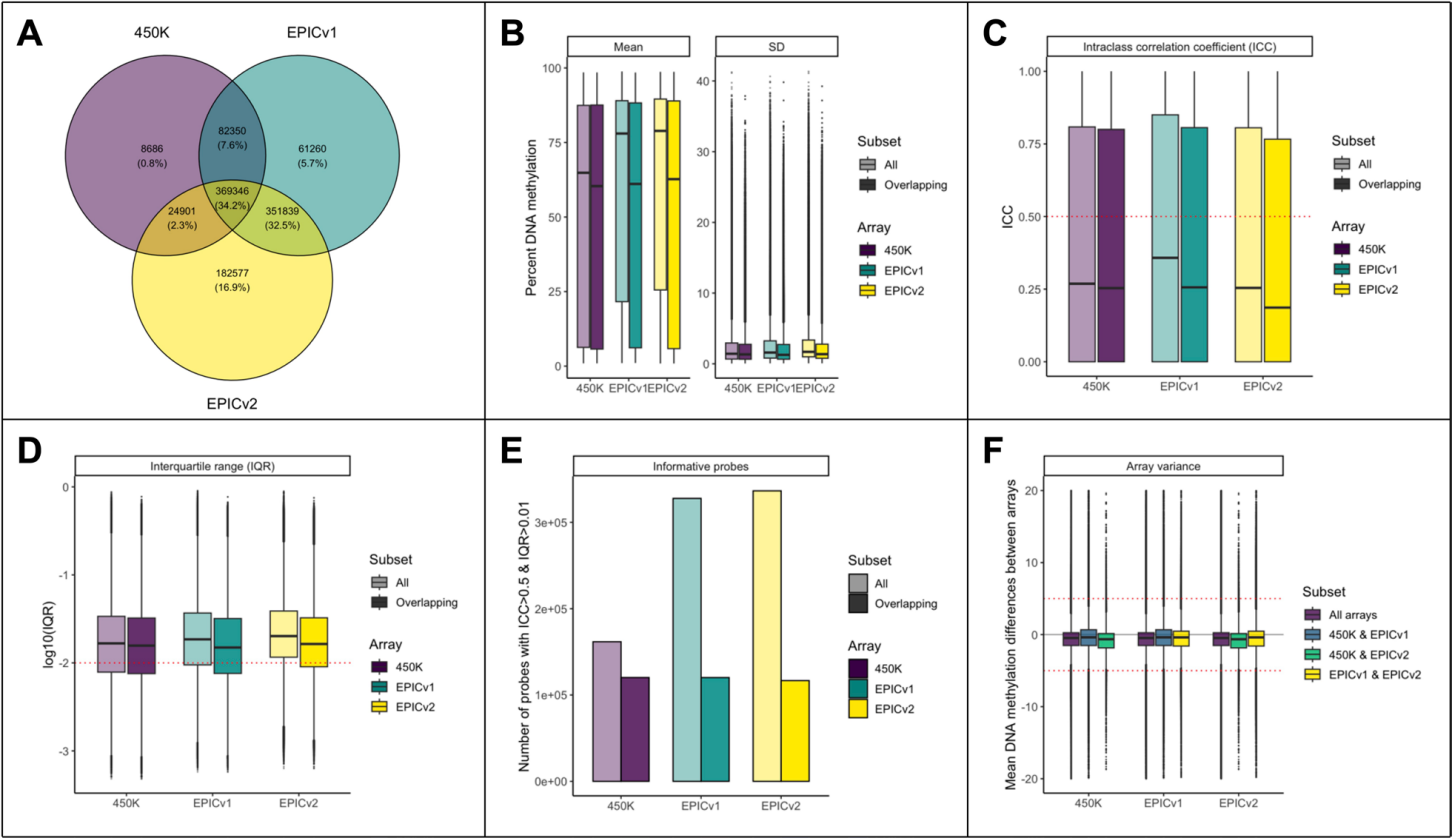
Summary of probe-level differences across arrays. **A** Representation of CpGs across array versions. **B** Mean and standard deviation (SD) of DNA methylation levels across CpGs for probes present on all three arrays. For each color set, the CpGs present on a given array are shown in the darker shade, while the CpGs present on all three arrays, dubbed overlapping, are shown in the lighter shade. **C** Intraclass correlations (ICC) for each CpG were calculated using sample replicates. An ICC > 0.5 is generally considered a good-quality probe (red dashed line) [33]. **D** Interquartile ranges of DNAm levels for each CpG are shown in − log_10_, where higher values represent better IQR. An IQR > 0.01 (red dashed line) was considered to capture meaningful biological variation between people. **E** Number of informative probes across each array (ICC > 0.5 and IQR > 0.01). **F** Mean difference in DNAm levels between arrays, as calculated from Tukey post hoc tests of array variance

Next, we gauged the quality of CpG probes across arrays found on the 450K, EPICv1, and EPICv2 arrays. Briefly, the EPICv2 introduces 184,259 new probes from the EPICv1, while reintroducing 24,597 probes from the 450K and losing 82,667 450K/EPICv1 probes (Fig. 1A). At the CpG-level, mean DNAm values and their standard deviations across all individuals on each array were slightly higher for probes on EPICv1 and EPICv2 compared to probes on the 450K array or that overlapped across all three arrays (Fig. 1B). Higher mean DNAm levels on the EPICv1 and EPICv2 were likely due to the addition of additional CpGs with higher mean DNAm (Figure S2). Individual-level DNAm levels were also marginally more variable on the EPICv1 (median SD = 1.59) and EPICv2 (median SD = 1.69) than the 450K (median SD = 1.42; Figure S3).

Probe-level intraclass correlations coefficients (ICC) varied across arrays, with slightly lower ICC values found on the 450K (median = 0.39) and EPICv2 (median = 0.38) arrays compared to EPICv1 (median = 0.42; Fig. 1C). Although these differences were small, they suggest that more probes measured on EPICv2 may be of lower reliability than those on EPICv1 and 450K. By contrast, EPICv2 probes had slightly higher interquartile range (IQR) values (median = 0.2) compared to the 450K (median = 0.17) and EPICv1 (median = 0.18) (Fig. 1D). When combining these two metrics to determine if CpGs were more likely to be informative (meaning an ICC > 0.5 and IQR > 0.01) [33], there was a net gain of 8,760 informative probes on the EPICv2 (336,460 total informative probes) compared to EPICv1 (327,700 total informative probes), though the proportion of informative probes on EPICv2 was lower (Fig. 1E).

Finally, we investigated whether DNAm levels were significantly different across arrays. Of the 828,436 CpGs present on at least two arrays, 642,205 (77.5%) showed significant differences between arrays, even after adjusting for multiple-test correction (FDR < 0.05). When estimating between-array differences using Tukey post hoc tests, the average DNAm difference between arrays was small (median = 0.98%; mean = 1.6%). Overall, 67,133 probes showed > 5% difference in DNAm levels between at least two arrays. Although these probes may be more influenced by array-based variation and thereby reflect less reliable DNAm measures, we note that many studies identify small differences in DNAm levels between groups (see for example [34–37]) and could therefore still be impacted by smaller array biases.

### Shiny application for CpG-level lookup

To provide access to the CpG-level annotations described above and further supplement their interpretation, we launched an R Shiny Application, the Cross-Array Comparison and Testing Interface (CACTI; https://cacti.geddes.rcac.purdue.edu/), which can be used to look up and download the statistics for specific CpGs. The information provided through this web portal includes CpG-level DNAm distributions, correlations across arrays, mQTL annotation from GoDMC [21], as well as detailed statistics of the array-based bias estimations.

### Concordance of sex associations across arrays

**Fig. 2:**
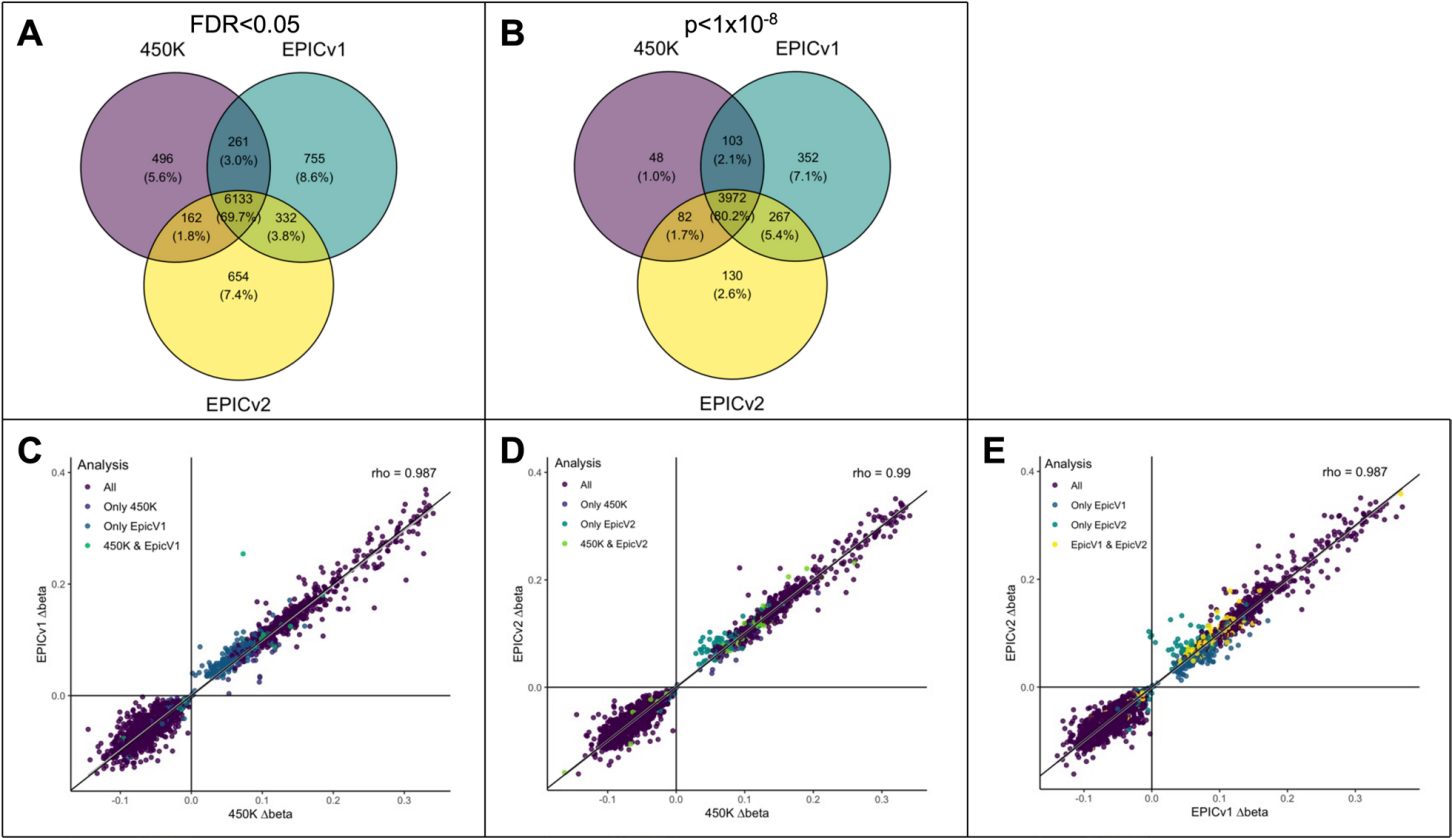
Summary of sex difference analyses. **A** Overlapping associations detected between DNAm and sex for each array (FDR < 0.05). **B** Overlapping associations detected between DNAm and sex for each array (*p* < 1 × 10^–8^). **C)** Comparison of mean DNAm differences between males and females (∆beta) for CpGs passing *p* < 1 × 10^–8^ (450K vs. EPICv1). **D** Comparison of mean DNAm differences between males and females (∆beta) for CpGs passing *p* < 1 × 10^–8^ (450K vs. EPICv2). **E** Comparison of mean DNAm differences between males and females (∆beta) for CpGs passing *p* < 1 × 10^–8^ (EPICv1 vs. EPICv2). Colors for CDE represent the array(s) on which the CpG met the significance threshold. Rho from Pearson correlations are shown

We next investigated if sex differences could be detected across arrays, by limiting our analyses to the 369,639 CpGs present on all three arrays to avoid detecting differences due to missing probes. In these analyses, 69.7% of associations were stable across all three arrays at an FDR < 0.05 (Fig. 2A; Table S3), which increased to 80.2% when using a more stringent *p* < 1 × 10^–8^ threshold (Fig. 2B). Replicated CpGs had higher IQR and standard deviation than CpGs associated with sex in single array types (Figure S4). Replicated CpGs also showed less variance in DNAm explained by array type, meaning that their DNAm levels were more stable across arrays. This finding suggests, array-based variance or bias may be a useful metric to gauge the reliability and reproducibility of results across array generations.

As p-values may be unreliable metrics for replication in epigenome-wide studies [35, 38, 39], we also assessed the concordance of effect estimates (i.e., difference in DNAm levels between males and females) across arrays. Overall, the effect estimates were very highly correlated across all arrays (*r* = 0.99; Fig. 2C–E). CpGs replicating more strongly across arrays also tended to have larger magnitudes of effect. Of the 4954 CpGs identified at a *p* < 1 × 10^–8^ (Table S3), only 3 (0.06%) showed discordant directions of effect across arrays. DNAm levels for these discordant CpGs were highly correlated between the 450K and EPICv2, but discordant for EPICv1, further pointing to the importance of assessing array-level differences when replicating results.

### Epigenetic clocks

Finally, we investigated the stability of epigenetic age estimations using current epigenetic clocks. In our dataset, the EPICv2 had more missing CpGs than the EPICv1 across all clocks, with missingness rates ranging from 3.5 to 32.6% (Table 2; Figure S5). CpGs missing on the EPICv1 accounted for 4.4% and 9% of the CpGs needed to derive the age estimate for Horvath1 and PhenoAge clocks, respectively. By contrast, the EPICv2 omits several additional CpGs across all epigenetic clocks, with the greatest impact on DunedinPoAm estimates (44.7% of CpGs needed for the estimate). While each clock had missing sites on the EPICv2, GrimAge1, GrimAge2, the Hannum clock, and DunedinPACE were most impacted (> 10% of CpG sites missing). Horvath1, Horvath2, and PhenoAge were less impacted by missing sites (< 4% missing).

**Table 2:** Summary of missing CpGs for different epigenetic clocks

Clock	Total CpGs	450K (%missing; % missing weight)	EPICv1 (% missing; % missing weight)	EPICv2 (% missing; % missing weight)
Horvath1	353	0	19 (5.4%; 4.4%)	13 (3.7%; 2.4%)
Horvath2	391	0	0	17 (4.35%; 3.5%)
Hannum	70	0	6 (8.6%; 9.0%)	7 (10.0%; 10.1%)
GrimAge1	1030	0	3 (0.3%; 0.0%)	185 (18.0%; 12.1%)
GrimAge2	1030	0	3 (0.3%; 0.0%)	185 (18.0%; 11.6%)
PhenoAge	513	1 (0.2%; 0.0%)	1 (0.2%; 0.0%)	18 (3.5%; 1.8%)
DunedinPoAm	46	0	0	15 (32.6%; 44.7%)
DunedinPACE	173	0	0	29 (16.8%; 11.4%)

**Fig. 3:**
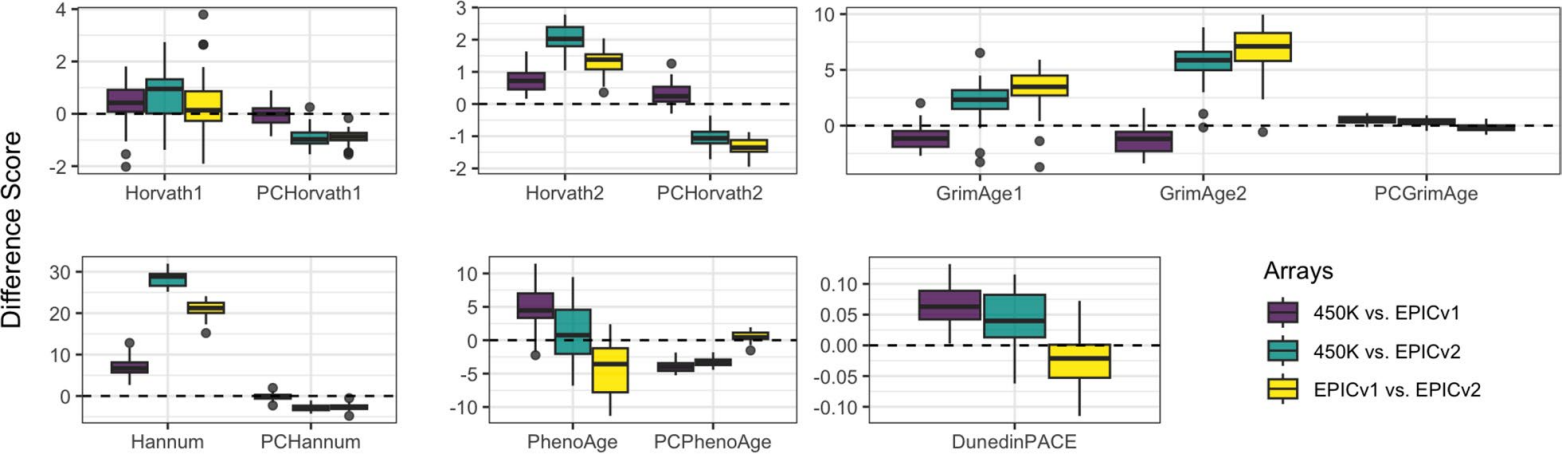
Pairwise comparison of epigenetic age estimates across arrays. Comparisons include the seven clocks included in the primary analyses, as well as the principal component (PC) versions of the clocks. The difference score (*y*-axis) represents the individual-level difference in epigenetic age estimate between two array versions, reading from left to right (e.g., 450K estimate minus EPICv1 estimate). For all clocks except DunedinPACE, the difference score reflects a difference in years of epigenetic age. For the DunedinPACE clock, the difference score reflects differences in the pace of aging

Overall, the average correlations of epigenetic age estimates between arrays decreased across generations (0.84 for 450K-EPICv1; 0.75 for 450K-EPICv2; and 0.71 for EPICv1-EPICv2) (Fig. 3; Figures S6–S7). In particular, GrimAge1 estimates showed considerably lower correlations for EPICv2 (*r* < 0.6 compared for 0.88 for 450K-EPICv1), while the Hannum clock consistently estimated negative ages for EPICv2 samples. The best performing clocks in the EPICv2 samples were DunedinPACE and Horvath2, which showed correlation above 0.78 with prior array generations and more accurate age estimations. In general, the EPICv2 underestimated epigenetic age compared to the other two arrays, a trend that was especially apparent for the Hannum, Horvath2, and GrimAge clocks (Fig. 3; Figure S6).

Importantly, the principal component versions of the clocks resulted in consistently strong correlations across all arrays (*r* > 0.97 across all clocks and arrays; Figure S8). In other words, principal component clocks effectively minimized technical noise, leading to more reliable epigenetic age measurements across array versions [31]. This finding suggests epigenetic age studies can benefit from principal component clocks, particularly when analyzing data from various Illumina arrays or focusing solely on newer ones.

## Discussion

The current study investigated how differences in DNAm measurements across the three latest generations of Illumina might influence epigenome-wide studies and epigenetic clock analyses. Three key findings emerged from this study, which have important implications for both longitudinal and clinical studies of DNAm patterns.

First, attempting to match samples across arrays based on SNP probes should be performed with caution, as a handful of SNP probes on the EPICv2 showed poor metrics, resulting in discordant genetic profiles when included in clustering. These differences could be due to differences in the sequence of the SNP probes, as well as poor calls resulting from color channel changes from the 450K and EPICv1 to the EPICv2 array. Such discrepancies pose challenges for studies relying on SNP probe matching to verify continuity in sample measurement over time [40]. SNP probe matching is particularly relevant in large, longitudinal cohort studies where the risk of sample misidentification is potentially higher. Therefore, we recommend removing the 21 problematic probes in future analyses to reduce any potential misleading sample matching or results.

Second, upon examining the reliability and stability of CpGs, we found widespread differences in the DNAm values measured across arrays, even at a very basic level. Beyond the fact that only ~ 370,000 probes are present across all three generations of arrays, a staggering 77.5% of these probes were significantly impacted by array-based variation. Although generally small in magnitude, these differences may be due to technical variation between arrays, either based on the position of probes or samples on the arrays (i.e., 12 samples/450K array versus 8 samples/EPIC array). These discrepancies can pose major problems to studies attempting to replicate or extend their findings using data from a different array or studies with multiple time points. For instance, longitudinal studies commonly move to newer arrays as they become available, leading to different time points being assessed with different arrays. This transition introduces a challenge in distinguishing between developmental changes and technical variability, especially when investigating time-varying associations with DNAm. For population-based studies, replicating data using a different array is also frequently impossible, as reprocessing samples for new arrays is limited by the quantity of available biological specimens. As such, our second recommendation is that researchers annotate their CpGs to gauge the stability of these sites across arrays. To this end, we created an online resource, the Cross-Array Comparison and Testing Interface (CACTI; https://cacti.geddes.rcac.purdue.edu/), which characterizes the concordance of DNAm values across arrays. CACTI also demonstrates how the quality of probes changed across platforms and how these changes can impact epigenome-wide association studies (EWAS). We also recommend researchers attempting to replicate or validate their findings using data from a different array consider the mean bias between arrays, as biases larger than effect estimates could mask replicated effects.

Finally, we advise studies employing different arrays consider PCA approaches when calculating epigenetic age as a method to enhance the reliability of these measures. Notably, our analyses of epigenetic clocks identified key differences when measuring epigenetic age on the EPICv2 array. Several clocks were missing impactful CpGs that accounted for up to 32.6% of the clock weights, resulting in large discrepancies in epigenetic age estimates compared to the 450K and EPICv1 arrays. This finding highlights an important and paradoxical trend toward losing important CpGs for epigenetic clocks in newer generations of Illumina arrays, despite the rising popularity of epigenetic clocks. While the transition from the 450K to EPICv1 resulted in relatively stable epigenetic age estimates [41], the recent transition to EPICv2 has had larger impact on their stability. Importantly, discrepancies in epigenetic age estimates were mitigated when using the principal component versions of the epigenetic clocks. As such, PCA approaches might be better suited to epigenetic age analyses using data from the EPICv2 array. Although the use of principal component versions of the epigenetic clocks may limit their interpretability, they are more likely to provide accurate and comparable estimates of epigenetic age, as current epigenetic clocks have been trained using 450K and EPICv1 data.

More broadly, our findings on the clocks may impact the interpretability of DNAm risk scores (or methylation profile scores), where multiple CpGs of small effect are aggregated into a single-value score [42–44]. Composite scores are increasingly being developed as a strategy to navigate the complexities posed by the high dimensionality of data, particularly in longitudinal studies aimed at tracking changes over time. These scores, including epigenetic clock estimates, are increasingly attractive to researchers since they provide a single value that can be analyzed as a predictor, outcome, or covariate, which is often more tractable than hundreds of thousands of CpGs. However, the change in arrays presents a considerable challenge to the replicability of DNAm risk scores, raising the question of whether the variability in DNAm risk scores reflects actual changes in development or represents an artifact of changes in the array technology used to measure them.

The present study has a number of noteworthy limitations. We used a relatively small sample of 30 participants (15 male; 15 female) with data across all three arrays, as well as 2–6 technical replicates across arrays. These small numbers may have influenced the ICC and IQR values of probes, resulting in a more stringent set of informative probes, as well as less robust set of association with sex. Second, our samples were primarily drawn from participants of African ancestry that originated from a small geographical area, which may limit the comparability to findings and data from other populations. We encourage studies in other under-represented ancestral groups to replicate our findings. Third, we analyzed blood samples from participants who were at the time around 5 years of age, which likely influenced the accuracy of epigenetic clock estimates, as most clocks are trained on adult participants. Similarly, most epigenetic clocks were generated from White individuals in Europe and North America, which would introduce additional bias into these analyses. While we anticipate array-based differences will be mostly stable across sample types (tissue, ages, ancestry, etc.), additional studies should replicate our results in datasets using DNAm measured in adults or other tissues. However, such replication efforts may be challenging, as Illumina no longer manufactures 450K and EpicV1 arrays and few studies will be able to derive concurrent DNAm measures from all three arrays across multiple participants.

## Conclusions

**Fig. 4:**
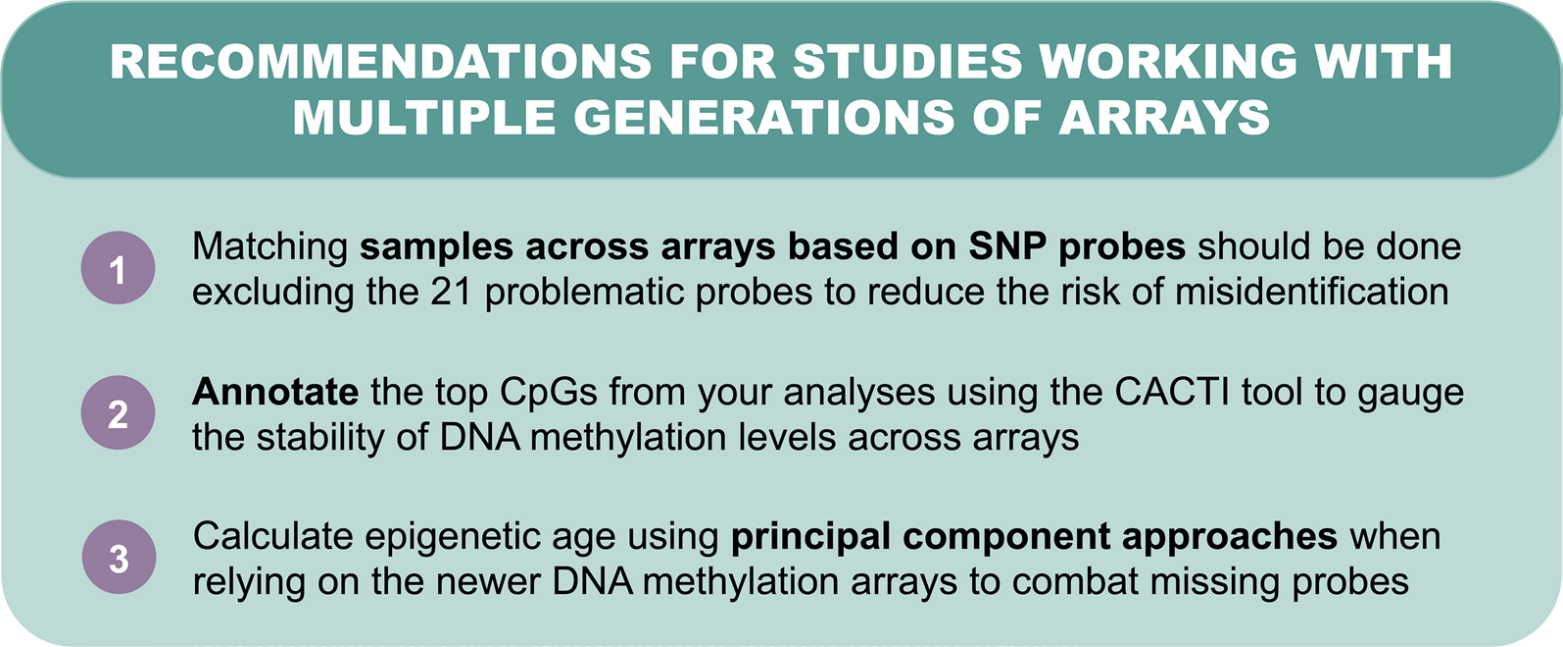
Recommendations for combining DNA methylation data across multiple generations of Illumina arrays. We provide three main recommendations for combining DNAm data across 450K, EPICv1, and EPICv2 arrays. Link to CACTI: https://cacti.geddes.rcac.purdue.edu/

In conclusion, we provide three practical recommendations to guide scientists in identifying SNP probes and creating epigenetic clocks across different arrays (Fig. 4). First, we encourage researchers matching their samples based on SNP probes exclude 21 of those probes in this process. Second, we encourage teams to review CACTI, a new resource to assess differences between three generations of the Illumina DNAm arrays, to understand how the reproducibility of associations may be influenced by differences in the stability and measurement of DNAm levels (https://cacti.geddes.rcac.purdue.edu/). Third, we suggest researchers consider the use of principal components when calculating epigenetic age using current clocks. Although the 450K and EPICv1 arrays have been phased out over the past years, they represent most epigenome-wide data currently available for analysis and replication. Given the importance of replicating findings in independent datasets, as well as investigating epigenetic changes across time in longitudinal studies, the CACTI will be crucial for future researchers to determine whether array-based differences influenced their findings. Ultimately, we hope findings from this study will push the field of epigenetics toward stronger inferences and more robust replication analyses that leverage the wealth of data generated from DNAm technologies.

## Supplementary Information

Supplementary Material 1.

Supplementary Material 2.

## Data Availability

The dataset analyzed during the current study is available from the Drakenstein Child Health Study upon appropriate approval. All original code used in this manuscript is available on Github: github.com/alussier17/alussier_scripts/tree/master/DNAm_array_versions. The full annotation of CpGs is available through our R Shiny application: https://cacti.geddes.rcac.purdue.edu/.
