# Supplementary material for: Technical variability across the 450K, EPICv1, and EPICv2 DNA methylation arrays: lessons learned for clinical and longitudinal studies
Source: Clin Epigenetics. 2024 Nov 22;16:166. doi: 10.1186/s13148-024-01761-4 (PMC11583407; doi:10.1186/s13148-024-01761-4)
Supplement: Supplementary file 1 — Supplementary Material 1. [file 13148_2024_1761_MOESM1_ESM.docx]

SUPPLEMENTAL TABLES

See excel file.

Table S1. Summary of male and female demographics

Table S2. SNP probe quality metrics.

Table S3. CpGs associated with sex in array-specific analyses of overlapping CpGs (p<1x10^-8^).

SUPPLEMENTAL FIGURES

| Figure S1. Clustering of individuals based on SNP profiles. |
| --- |
| 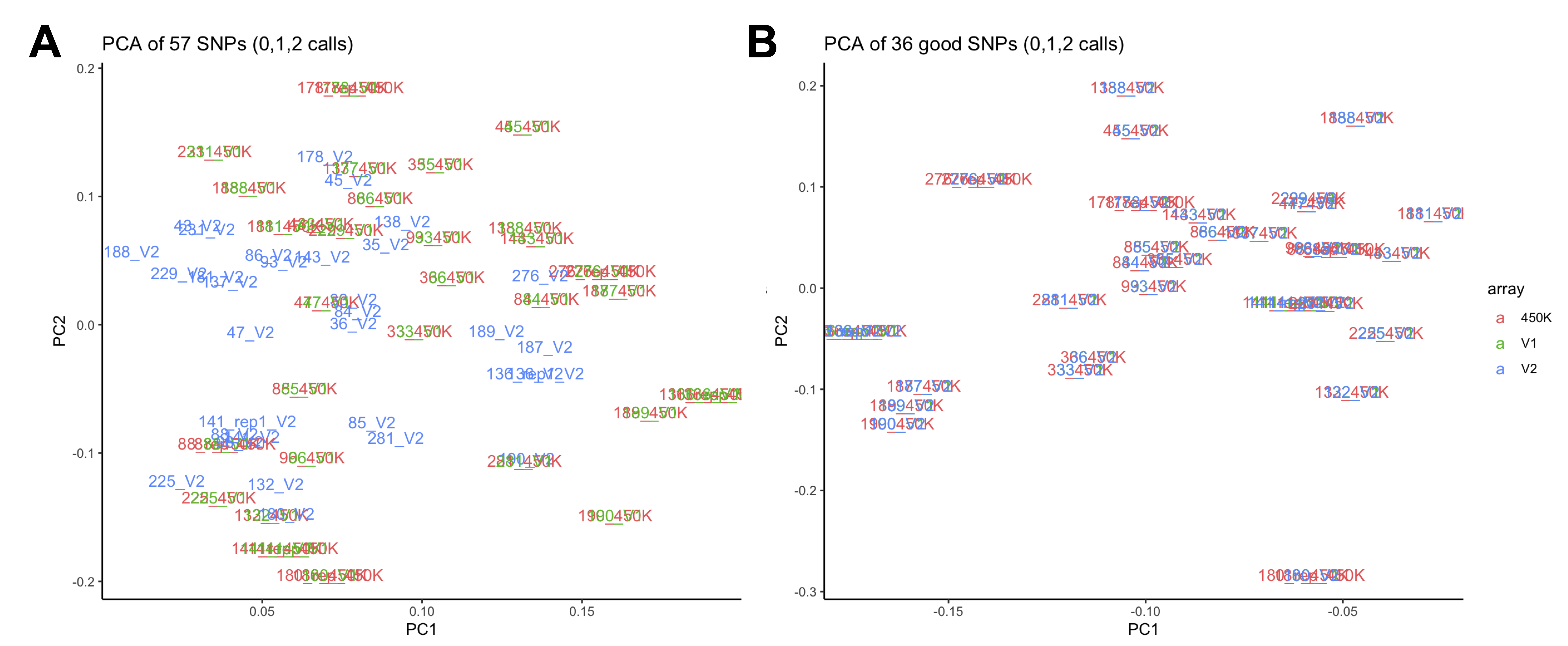 |
| The plots show the two first principal components (PC) of SNP profiles for each sample, noted as sampleID_array.  **A)** PCs estimates from the 57 SNPs present on all three arrays show discrepancies for the EPICv2 samples (blue) compared to the 450K (red) and EPICv1 (green) samples.  **B)** After removing SNP probes with poor quality metrics, the EPICv2 samples cluster perfectly with their 450K and EPICv1 counterparts. |

| **Figure S2. Density plot of mean DNA methylation levels across arrays** |
| --- |
| 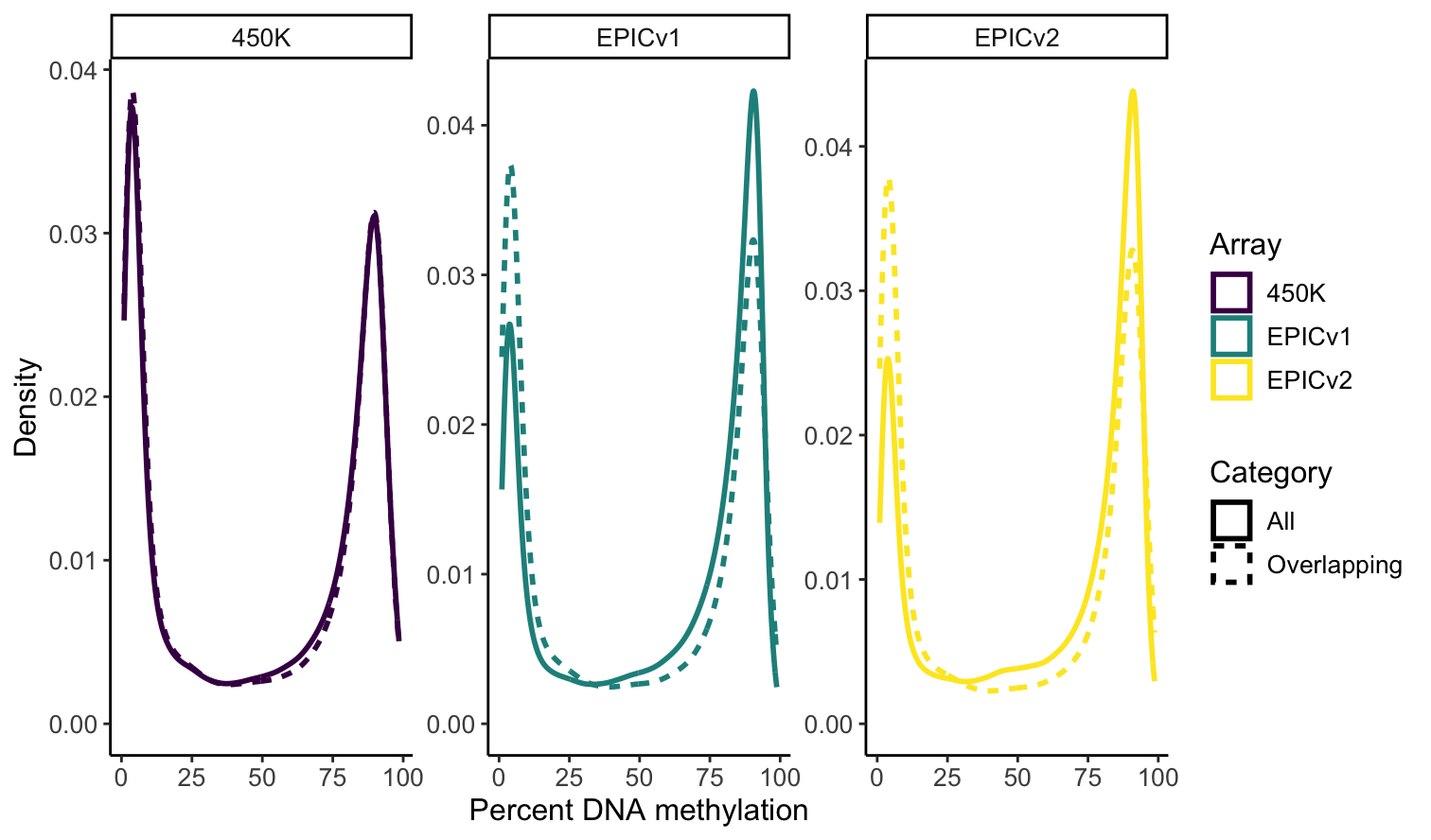 |
| Density plots of the mean DNA methylation levels for CpGs present on the different arrays. Dotted lines show the CpGs that were present across all three arrays. |

| **Figure S3. Zoom-in of the CpG-level standard deviations** |
| --- |
| 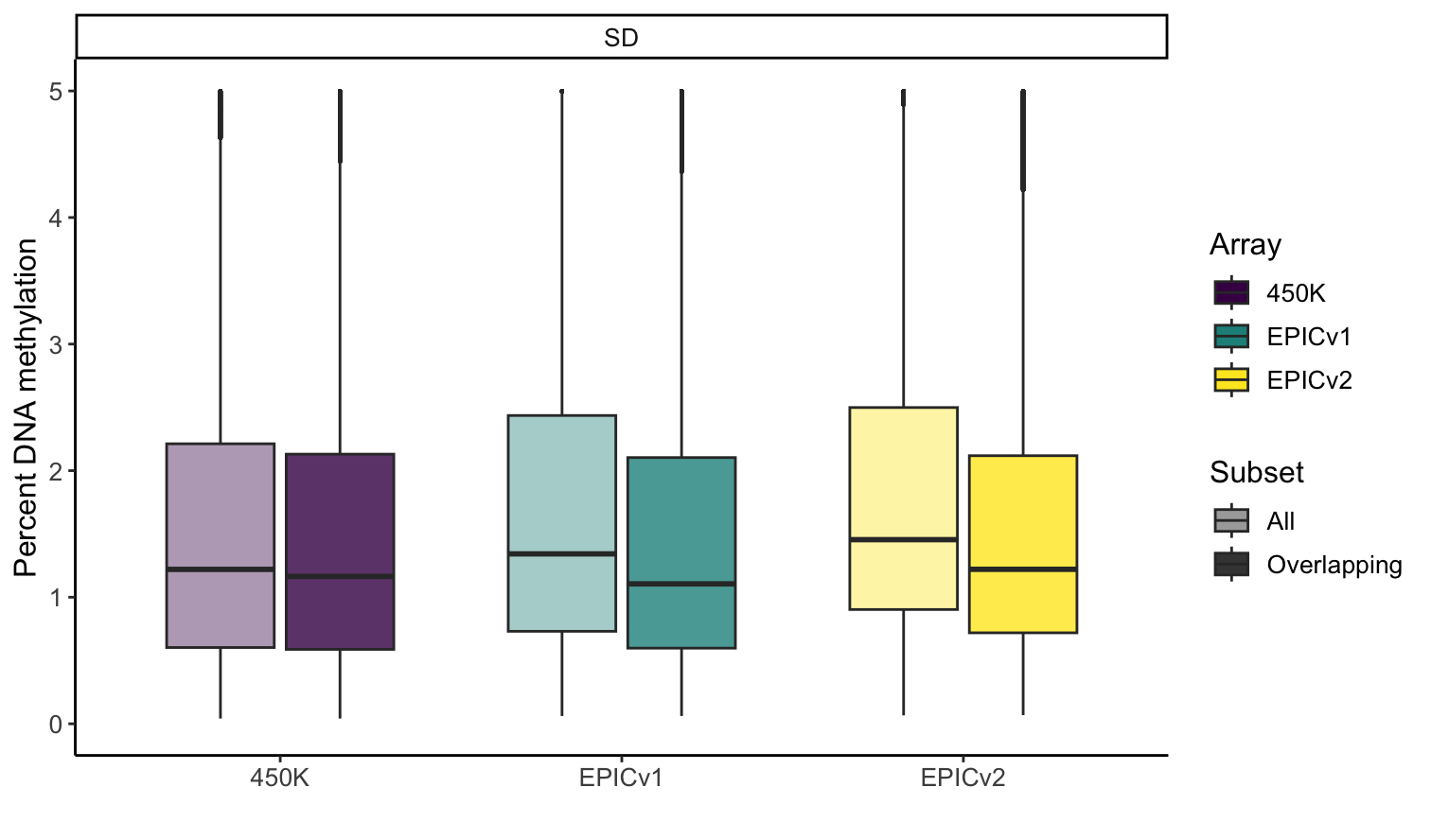 |
| Standard deviation (SD) of DNA methylation levels across CpGs for probes present on all three arrays. For each color set, the CpGs present on a given array are shown in the darker shade, while the CpGs present on all three arrays, dubbed overlapping, are shown in the lighter shade. This figure has been zoomed-in from Figure 1B to show CpGs with a SD below 5% of DNA methylation to provide a better representation of the marginal differences between arrays (450K median SD=1.42; EPICv1 median SD=1.59; median SD=1.69). |

| **Figure S4. Annotation of CpGs from the array-level EWAS of sex differences.** |
| --- |
| 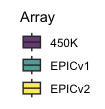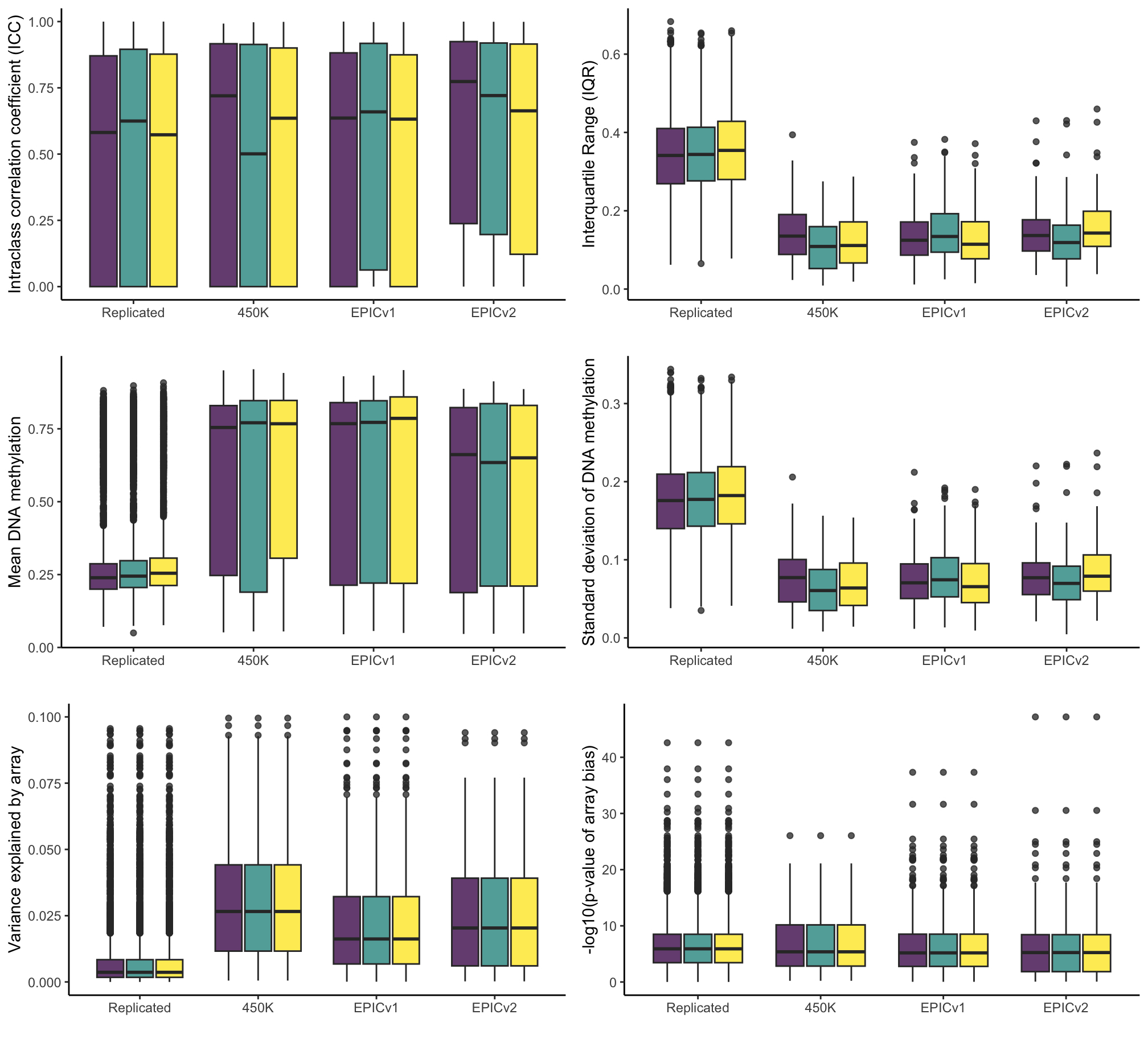 |
| Primary characteristics of the CpGs meeting a p<1x10^-8^ threshold in the epigenome-wide association studies (EWAS) of sex differences. We show CpGs that were replicated across all three arrays (“Replicated”; 3,530 CpGs), as well as the CpGs associated with sex on specific arrays only (450K = 17 CpGs; EPICv1 = 390 CpGs; EPICv2 = 152 CpGs). Colors represent the values on each of the three arrays (450K = purple; EPICv1 = green; EPICv2 = yellow). |

| Figure S5. Weighting of missing CpGs across arrays and epigenetic clocks. |
| --- |
| 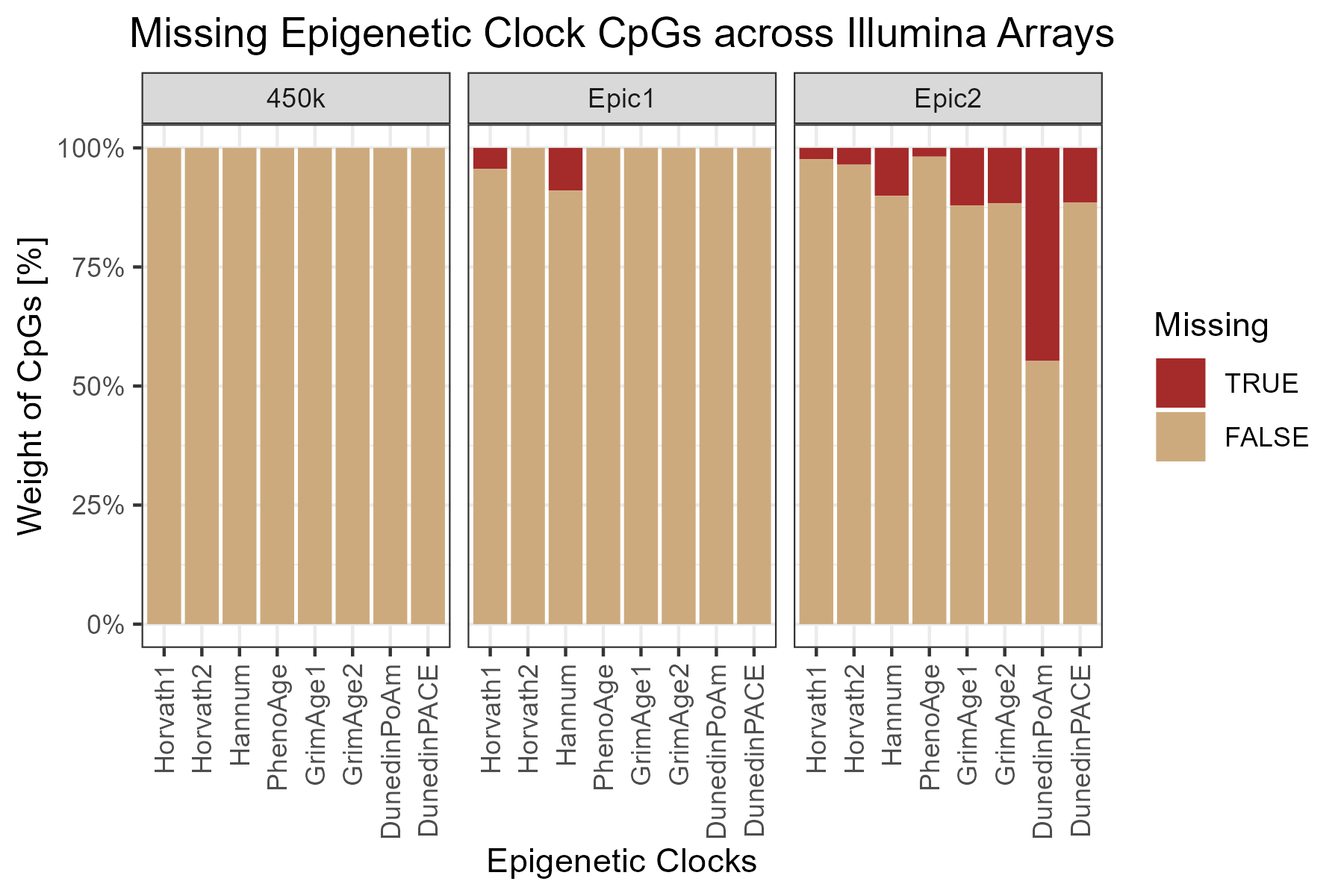 |
| For each clock, we extracted the set of CpG sites along with their corresponding weights from the different epigenetic age algorithms. Most epigenetic clocks compute epigenetic age through a one-step approach, involving the weighting of DNA methylation levels across a set of CpG sites. By contrast, the GrimAge clocks apply a two-step approach, generating a series of estimates based on a set of weighted CpG sites that are subsequently weighted again to form a singular epigenetic age measure. For comparison of the weights of absent CpG sites between one- and two-step approaches, we normalized GrimAge CpG weights by multiplying the absolute values of first step and second step weights for each CpG site. |

| Figure S6. Concordance of epigenetic age estimates across clocks and arrays. |
| --- |
| 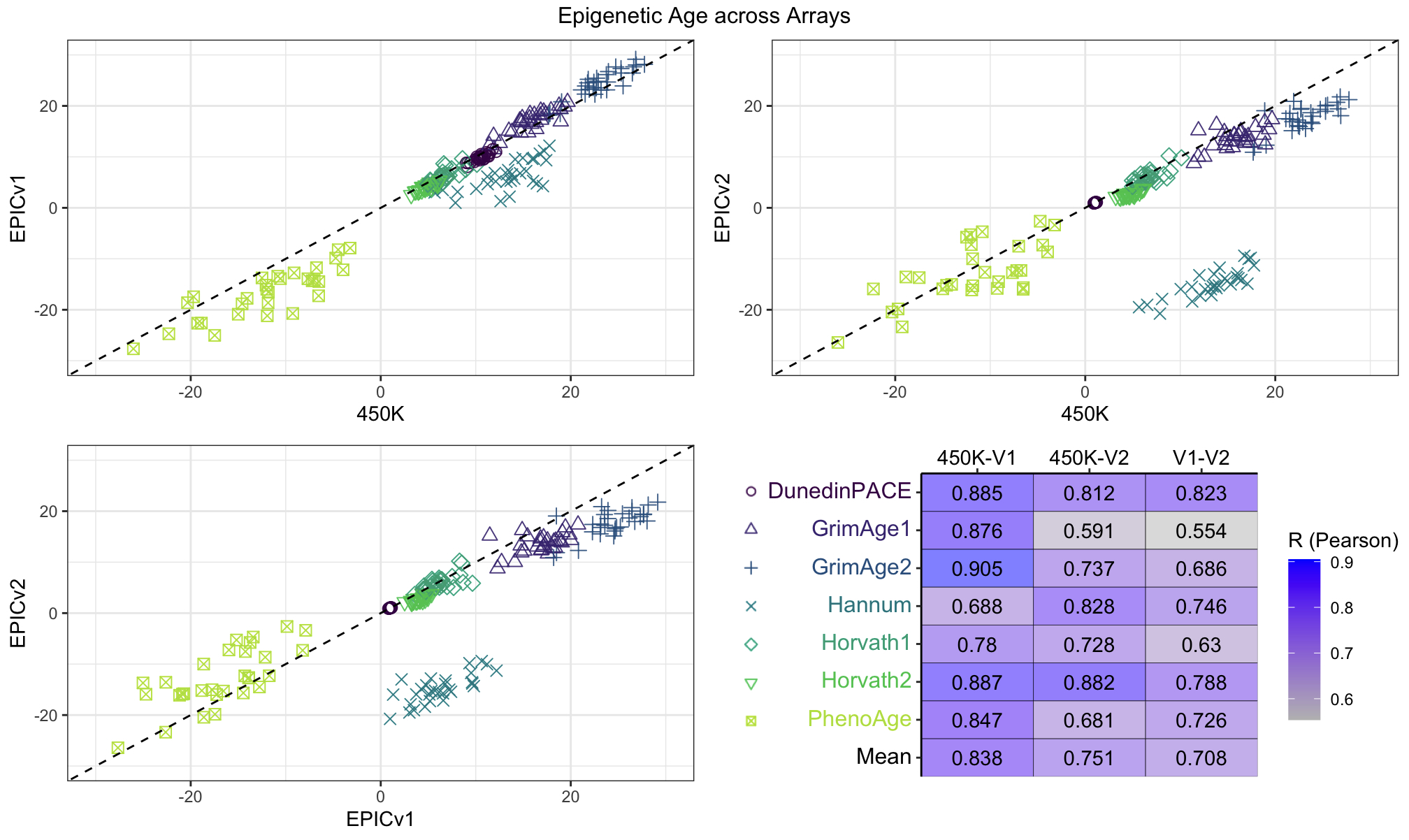 |
| Comparison of the epigenetic age estimates for all epigenetic clocks. Colors and shapes refer to the different epigenetic clocks, with Pearson correlations shown for each array comparison. The mean across all clocks is also shown and highlights an overall decrease in the replicability of epigenetic age estimates in newer arrays. |

| Figure S7. DunedinPACE epigenetic age estimation across arrays. |
| --- |
| 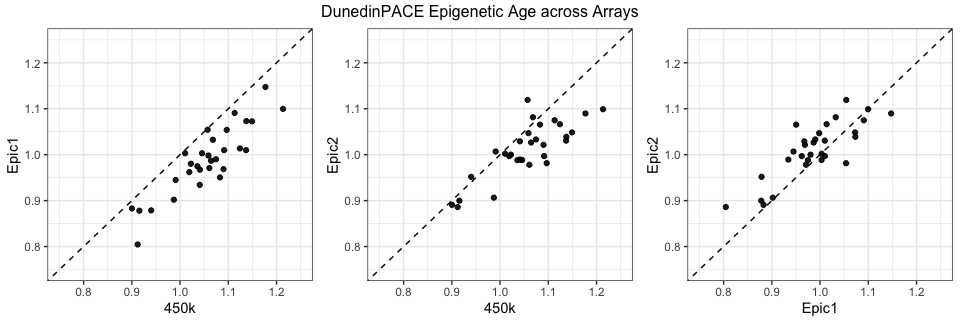 |
| Comparison of epigenetic age estimates across arrays for the DunedinPACE clock. These results are shown separately from Figure S5 due to the small numbers. |

| Figure S8. Concordance of epigenetic age estimates across principal component clocks and arrays. |
| --- |
| 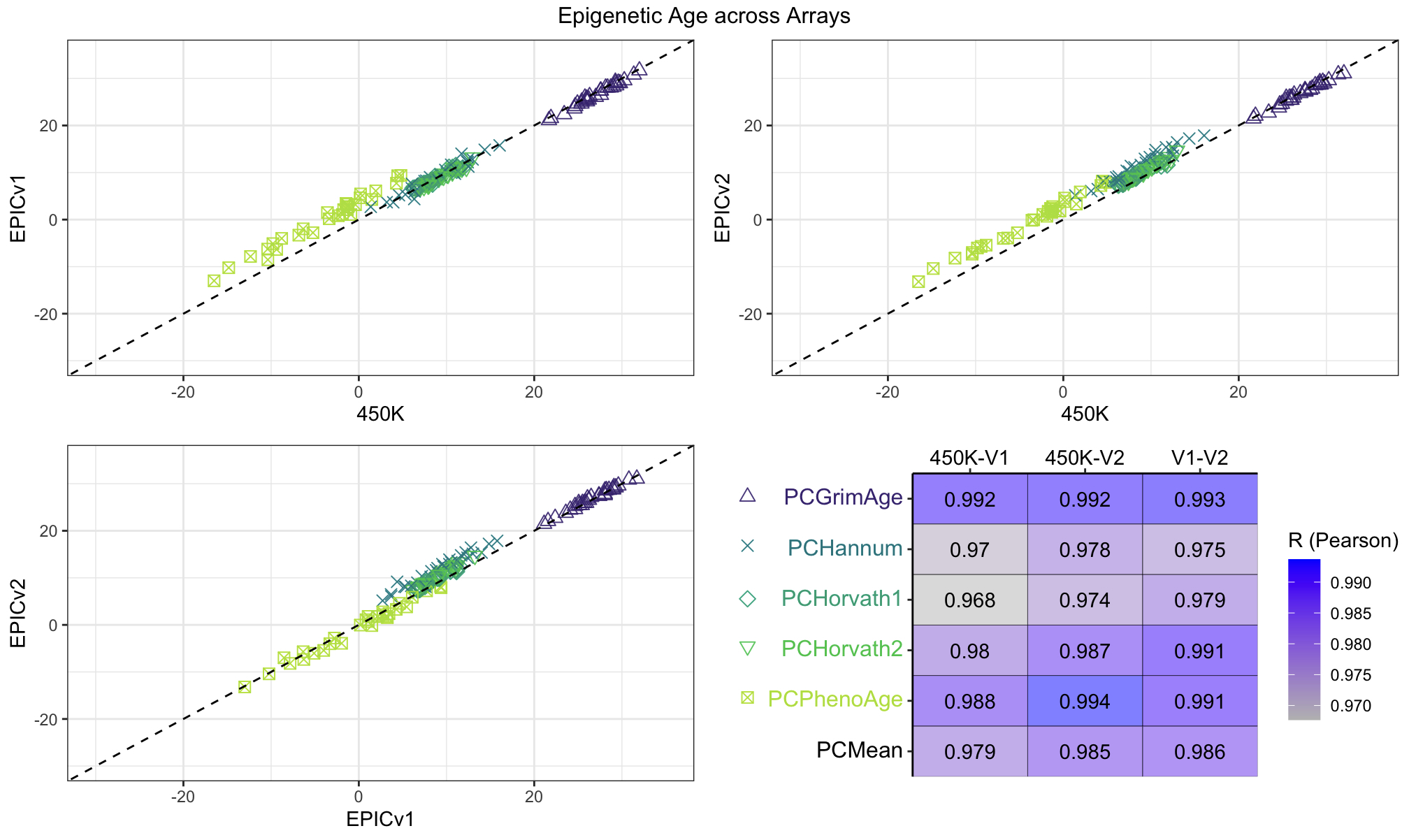 |
| Comparison of the epigenetic age estimates for all epigenetic clocks using principal components (PC). Colors refer to the different epigenetic clocks, with Pearson correlations shown for each array comparison. The PC versions of the GrimAge2 and DunedinPACE clocks were unavailable. |
